# Food shopping behaviors of residents in two Bronx neighborhoods

**DOI:** 10.3934/publichealth.2016.1.1

**Published:** 2015-12-24

**Authors:** Rachel Dannefer, Tamar Adjoian, Chantelle Brathwaite, Rhonda Walsh

**Affiliations:** 1 Bureau of Chronic Disease Prevention and Tobacco Control, New York City Department of Health and Mental Hygiene; 2 Center for Health Equity, New York City Department of Health and Mental Hygiene

**Keywords:** food access, food environment, nutrition, health disparities, supermarkets, corner stores, New York City

## Abstract

**Background:**

Numerous researchers have documented associations between neighborhood food environments and residents' diets. However, few quantitative studies have examined the food shopping behaviors of residents in low-income neighborhoods, including the types of stores patronized and frequency of visits. This study presents findings on the food shopping behaviors of residents in the Bronx neighborhoods of West Farms and Fordham.

**Methods:**

Street-intercept surveys were conducted in spring 2012 with residents of West Farms and Fordham as part of a broader program evaluation. The survey included questions on general food shopping behaviors including visits to neighborhood bodegas (corner stores) and supermarkets, mode of transportation to the supermarket most commonly frequented, and the primary source for purchases of fruits and vegetables.

**Results:**

The survey was conducted with 505 respondents. The sample was 59% Hispanic and 34% black, with a median age of 45 years. Thirty-four percent of respondents had less than a high school education, 30% were high school graduates or had their GED, and 36% had attended some college. Almost all respondents (97%) shopped at supermarkets in their neighborhood; 84% usually shopped at a supermarket within their neighborhood, and 16% usually shopped at a supermarket outside of their neighborhood. Most respondents (95%) shopped at bodegas in their neighborhood, and 65% did so once per day or more.

**Conclusions:**

Residents of these neighborhoods have high exposure to local food stores, with the vast majority of respondents shopping at neighborhood supermarkets and bodegas and almost 2 in 3 respondents going to bodegas every day. These findings demonstrate the important role of supermarkets and bodegas in local residents' shopping patterns and support the inclusion of these stores in efforts to create food environments that support and promote healthy eating.

## 1. Introduction

Concerns about increasing rates of obesity and other diet-related diseases have led researchers and practitioners to examine the relationship between food environments and residents' diets. Several studies have found associations between local food stores and diet, as well as neighborhood disparities in the availability of healthy food based on race/ethnicity and income[1],[2]. A number of strategies have been implemented across the country to improve food environments, including providing incentives for new supermarkets to locate in underserved neighborhoods and encouraging corner stores to carry and promote healthier foods[2],[3]. Despite the substantial literature demonstrating relationships among food deserts, poor dietary patterns, and obesity, there has been little quantitative evidence confirming how neighborhood residents interact with their food environments[1]. Qualitative research on food shopping behavior has found that a number of factors influence consumer choices. Several studies have found that shoppers favored stores that were closest to their homes or workplaces, offered the most affordable merchandise, and/or offered the best variety or quality of products[4]–[6]. However, limited focus has been placed on understanding the food shopping behaviors of residents in low-income neighborhoods, including the types of stores patronized and frequency of visits. This paper aims to contribute to the body of research on local residents' shopping behaviors at neighborhood food stores.

The New York City (NYC) Department of Health and Mental Hygiene (Health Department) explored food shopping behaviors among residents of West Farms and Fordham, two adjacent neighborhoods located in the borough of the Bronx in NYC. From 2012 to 2013, the Health Department implemented a program called Shop Healthy NYC (Shop Healthy) in these neighborhoods in order to increase access to and consumption of healthier food. The Shop Healthy model builds on lessons learned through almost a decade of Health Department programs with food retailers in low-income neighborhoods, including work with more than 50 supermarkets and 1,000 bodegas. Bodegas, also referred to as corner stores, are common throughout NYC and carry a range of mostly non-perishable products. Shop Healthy's approach is based on the social-ecological model, which recognizes that individuals interact with multiple levels of influence, including societal, community, institutional, interpersonal, and individual, and illustrates the need to intervene at all of these levels[7]. Following this model, Shop Healthy employs a comprehensive approach to food retail by addressing multiple components of the food environment. The initiative develops relationships with supermarkets, bodegas, and food suppliers/distributors to improve placement, promotion, and availability of healthier products, while providing tools and technical assistance to community members and partners to increase demand for healthier foods and support participating retailers[7]. As part of the evaluation of Shop Healthy in West Farms and Fordham, street-intercept surveys were conducted with residents of these neighborhoods[8]. This paper reports on survey findings relating to respondents' food shopping behaviors.

## 2. Materials and methods

This project was reviewed by the DOHMH Institutional Review Board (IRB) and was determined to be exempt from IRB oversight as it constituted a public health program evaluation.

### 2.1. Setting

**Figure 1.**
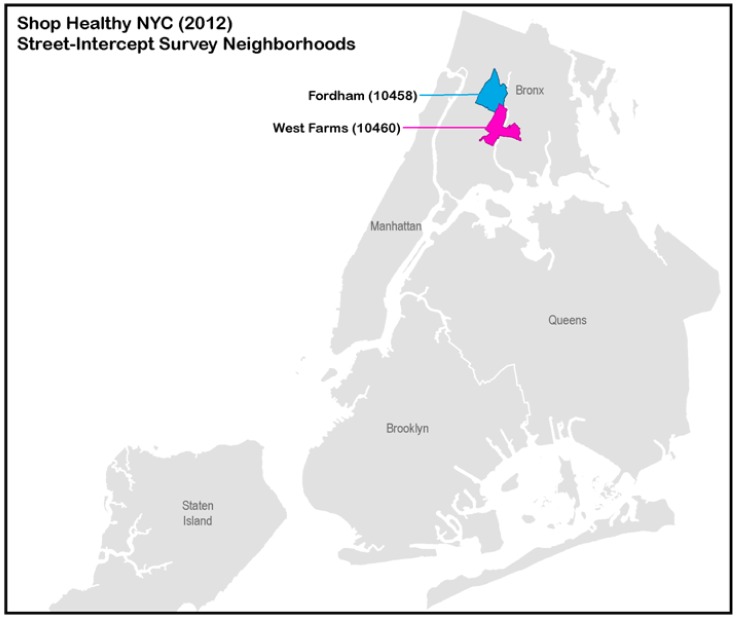
Shop Healthy NYC (2012), Street-Intercept Survey Neighborhoods.

The Bronx is the northernmost of NYC's five boroughs (Figure 1), each of which constitutes a county. Among all 62 counties in New York State, the Bronx ranks lowest in terms of health outcomes, income, and educational attainment[9]. The South Bronx, where West Farms is located, and Fordham/Bronx Park, where Fordham is located, have significantly higher proportions of residents that are overweight or obese than the rest of the city (71% in the South Bronx, 72% in Fordham/Bronx Park, and 55% in the rest of NYC). These neighborhoods also have a high prevalence of diabetes, with 16% in the South Bronx and 15% in Fordham/Bronx Park compared to 10% in the rest of NYC[10]. The West Farms and Fordham ZIP codes also have high rates of poverty. Forty-one perecent of the 56,084 West Farms residents and 38% of the 74,859 Fordham residents fall below the federal poverty level, compared with 20% citywide. About two-thirds of residents have a high school education or less (68% in West Farms, 64% in Fordham) compared with 45% citywide. The majority of residents are Hispanic/Latino (71% in West Farms, 64% in Fordham), followed by non-Hispanic black (24% in West Farms, 19% in Fordham)[11]. In 2012 there were more than 10 bodegas for every supermarket in West Farms and Fordham, with 189 bodegas and 18 supermarkets in total[8].

### 2.2. Survey implementation

In April 2012, street-intercept surveys were conducted with residents of West Farms (ZIP code 10460) and Fordham (ZIP code 10458) in order to assess the community-level impact of implementing Shop Healthy in these neighborhoods. The street-intercept method can be more effective in obtaining a representative sample among low-income populations than more traditional sampling strategies[12]. Pairs of interviewers were stationed at five locations per ZIP code including one transportation hub, one library, and three areas with a high concentration of food retail outlets. The target sample size was 500 surveys, with 50 per location. Interviewers received a 2-hour classroom training followed by field training. To recruit respondents, interviewers asked passersby if they would be willing to complete a short survey about food in the neighborhood, for which they would receive a single-fare public transit pass, valued at $2.25, as an incentive. Respondents were required to be 18 years of age or older and to live in either West Farms or Fordham. Consent was not obtained as the survey was anonymous and collected no identifying information. Surveys were conducted in English and Spanish on Mondays through Saturdays from 8 am to 11 am, 12 pm to 3 pm, and 4 pm to 7 pm.

### 2.3. Measures

The measures used for our analyses are described below. Several questions were developed by the Health Department specifically for this project or adapted from previously developed internal evaluation tools. Additional questions were from publicly available surveys as cited below. The survey was pretested for clarity and length, revised accordingly, and pretested again before being finalized.

Questions about supermarket shopping included how often respondents shopped at neighborhood supermarkets, how often they purchased fruits and vegetables at a neighborhood supermarket, whether their usual supermarket was within or outside of their neighborhood, and the mode of transportation and travel time to their usual supermarket. Questions on bodega shopping included how often respondents shopped at neighborhood bodegas and how many blocks the bodega they visited most often was from their home. A question was taken from the NYC Community Health Survey on how many servings of fruits and vegetables the respondent consumed on the previous day. Two separate NYC Community Health Survey questions on consumption of soda and consumption of other sweetened drinks were combined to create one question on how often the respondent drank sugary drinks like soda, sweetened iced tea, sports drinks, fruit punch or other fruit-flavored drinks[10]. Respondents were asked an open-ended question about what they thought could be done to improve the eating habits of people in their neighborhood, which was adapted from a health-related survey from the office of the Bronx Borough President[13]. For this question, several response options were listed, and interviewers were instructed to select all options that applied based on the respondent's answer. Finally, respondents were asked several demographic questions including their residential ZIP code, gender, age, race/ethnicity, and education level. Educational level reported in this paper was restricted to those 25 years and up, consistent with the US Census and NYC Community Health Survey methodology[10],[11].

### 2.4. Analysis

Descriptive statistics were used to summarize respondents' shopping behaviors, opinions on improving neighborhood eating habits, and demographic characteristics. Bivariate significance testing was used to assess associations between respondent demographics and shopping behaviors and to explore whether daily versus non-daily bodega shoppers differed in terms of consumption of fruits and vegetables and sugary drinks. All analyses were conducted with SPSS version 18.0 (SPSS, Inc., Chicago) with α = .05.

## 3. Results

A total of 505 baseline surveys were collected, with a 40% response rate. The median respondent age was 45 years, with a range of 18 to 87. The sample was 53% female, 59% Hispanic, and 34% black. Thirty-four percent of respondents had less than a high school education, 30% were high school graduates or had their GED, and 36% had attended some college. Forty-seven percent lived in the Fordham ZIP code and 53% lived in West Farms (Table 1).

**Table 1.** Characteristics of Shop Healthy NYC Street-intercept Survey Respondents (N = 505), Bronx, NY, 2012

	N (%)	95% CI
**Residential ZIP code**		
10458 (Fordham)	239 (47.3)	(43.0, 51.7)
10460 (West Farms)	266 (52.7)	(48.3, 57.0)
**Gender**		
Male	234 (46.5)	(42.1, 50.1)
Female	267 (53.1)	(48.7, 57.5)
**Age Group**		
18–24	55 (10.9)	(8.2, 13.6)
25–44	189 (37.4)	(33.2, 41.7)
45–64	229 (45.3)	(41.0, 49.7)
65+	32 (6.3)	(4.2, 8.5)
**Race/Ethnicity**		
Hispanic	294 (59.2)	(54.8, 63.5)
Black	169 (34.0)	(29.8, 38.2)
White	18 (3.6)	(2.0, 5.3)
Other	16 (3.2)	(1.7, 4.8)
Education*		
Less than HS	153 (34.3)	(29.9, 38.7)
HS Graduate/GED	132 (29.6)	(25.3, 33.8)
Any College	161 (36.1)	(31.6, 40.6)

*Education is presented among those aged 25 and up

Almost all respondents shopped at supermarkets in their neighborhood (97%), and most (60%) did so once per week or more (Table 2). Overall, 83% of respondents walked to their usual supermarket and 16% drove a personal car or used public transportation. However, transportation varied based on whether respondents usually shopped within or outside of their neighborhood. Among respondents (84%) who most often shopped at a supermarket *within* their neighborhood, 94% walked to their usual supermarket with a mean travel time of 7 minutes. A notable minority of respondents (16%) usually shopped at a supermarket *outside* of their neighborhood. Among these respondents, the most common methods of transportation to their usual supermarket were personal car (43%), bus (26%), and walking (23%), with an overall mean travel time of 19 minutes (Table 3). Notably, of the respondents whose usual supermarket was outside of their neighborhood, 91% still shopped at supermarkets in their neighborhoods on occasion, and 93% shopped at neighborhood bodegas (data not shown). The majority of respondents (76%) bought most of their fresh produce from supermarkets (Table 2); these respondents reported buying produce at their supermarket 1.4 times per week on average (data not shown).

**Table 2.** Respondent Shopping Behaviors, Shop Healthy NYC Street-intercept Survey (N = 505), Bronx, NY, 2012

	N	%
**Supermarket Shopping**		
Ever shops at neighborhood supermarkets	485	96.8
Frequency of shopping at neighborhood supermarkets		
Never	16	3.2
Less than once per week	184	36.7
Once per week or more	301	60.1
Usual supermarket is within respondents' neighborhood	413	83.8
Usual supermarket is outside respondents' neighborhood	80	16.2
**Bodega Shopping**		
Ever shop at neighborhood bodegas	477	94.6
Frequency of shopping at neighborhood bodegas		
Never	27	5.4
1-2 times per month	5	1.0
1-6 times per week	142	28.2
Once per day or more	330	65.5
**Fruit and Vegetable Purchasing**		
Most common source for fruit and vegetable purchases		
Supermarket	376	75.8
Fruit and vegetable store	51	10.3
Green Cart (sell fresh produce in areas with limited access to healthy foods) or other fruit & vegetable cart	28	5.6
Bodega	23	4.6
Farmers' Market	5	1.0
Other	13	2.6

The vast majority (95%) of respondents shopped at bodegas in their neighborhood and 65% visited a neighborhood bodega once per day or more (Table 2). Among respondents who shopped at neighborhood bodegas, the store visited most often was an average of 1.4 blocks from their home (data not shown).

Most supermarket and bodega shopping behaviors did not vary significantly by demographic subcategories. There were no significant differences by gender, age group, race/ethnicity, or educational attainment of shoppers who primarily patronized a supermarket within their neighborhood, or most often purchased fruits and vegetables at supermarkets or other locations. We did find that as age increased, a lower percentage of respondents reported shopping at bodegas daily. Among respondents aged 65 and older, 53% were daily shoppers, compared with 84% among ages 18–24. Additionally, a higher percentage of men reported daily bodega shopping than women (72% vs. 59%, *p* < 0.01) (Table 4). Daily bodega shoppers reported consuming significantly more sugary drinks per week compared with other shoppers (10.9 vs. 4.6, *p* <.001) (data not shown).

In response to the question on what could be done to improve eating habits of neighborhood residents, the top three responses were “nutrition education” (26%), “improved access to quality produce” (23%), and “more affordable healthy foods” (21%) (data not shown).

**Table 3.** Respondent Mode of Travel to Usual Supermarket, Shop Healthy NYC Street-Intercept Survey (N = 493)*, Bronx, NY, 2012

	Overall		Location of Usual Supermarket			
			Within Neighborhood		Outside Neighborhood	
	N(%)	Mean time (min)	N(%)	Mean time (min)	N(%)	Mean time (min)
Overall	493 (100.0)	9.1	413 (100.0)	7.3	80 (100.0)	18.6
Mode of travel to usual supermarket						
Walk	408 (82.8)	7.6	390 (94.4)	7.1	18 (22.5)	18.1
Personal Car	45 (9.1)	14.4	11 (2.7)	7.7	34 (42.5)	16.5
Bus	29 (5.9)	19.7	8 (1.9)	14.4	21 (26.3)	21.7
Cab	6 (1.2)	16.7	2 (0.5)	12.5	4 (5.0)	18.8
Train	4 (0.8)	20.0	1 (0.2)	5.0	3 (3.8)	25.0

*missing data not reported

## 4. Discussion

The large majority of residents surveyed in the Bronx communities of West Farms and Fordham have high exposure to the neighborhood food environment through frequent trips to local supermarkets and bodegas. We found that 97% of respondents shopped at neighborhood supermarkets, with 83% walking to their usual supermarket and 16% driving or using a bus or train. These corroborate results from a study in the Bronx neighborhood of Morrisania, which found that 94% of respondents shopped at a local supermarket or discount store, and that most people (77%) walked to their usual store and 11% drove or used public transportation[12]. This pattern contrasts with other cities, including Seattle and Philadelphia, where researchers have noted that most people use their cars for food shopping[16],[17]. This points to the place-based nature of shopping behaviors and highlights the need for approaches which are tailored to local community practices.

Exposure to bodegas was high, with 95% of all respondents shopping at neighborhood bodegas and 65% going to bodegas on a daily basis. These findings are consistent with related studies conducted in NYC. Surveys with bodega shoppers at 171 stores throughout NYC showed that 71% of respondents shopped at bodegas five or more times per week[18]. Another study which surveyed shoppers at four bodegas in the South and Central Bronx reported that 68% of shoppers went to bodegas once per day or more[3].

Our findings demonstrate the important role of supermarkets and bodegas in residents' shopping patterns and support the inclusion of these stores in efforts to create food environments that support and promote healthy eating. Even among respondents whose usual supermarket was outside their neighborhood, the majority still shopped at neighborhood supermarkets and bodegas. Given research showing that people most often use bodegas for purchasing convenience items such as beverages and snacks and do the majority of their grocery shopping at supermarkets[3],[12],[17],[18], retail-based interventions should consider strategies that reflect how people use different types of stores. The evolution of the NYC Health Department's own retail-focused work reflects this learning. While many of the agency's early efforts encouraged bodegas to expand produce offerings, the focus has since shifted to instead encourage bodegas to offer and promote healthier convenience items such as water, healthier snacks, and healthier deli options. Additionally, the agency broadened its food retail work to include established supermarkets. Although supermarkets have many healthy options, they also tend to offer and promote a wide range of unhealthy products[19]. Recent studies questioning whether proximity to supermarkets is sufficient to change diets points to the importance of ensuring that the supermarkets are not only present in a neighborhood but also promote high-quality and healthy foods[12].

We found that daily bodega shoppers were younger, more likely to be male, and consumed more sugary beverages than non-daily shoppers. Other studies have found sugary drinks to be among the top 3 items purchased from bodegas, with 18% to 31% of customers purchasing sugary drinks at the visit when they were surveyed[3],[18]. Research shows that sugary drink consumption is significantly correlated with population-level overweight and obesity rates[20], and that regular consumption of sugary drinks is also associated with increased risk of chronic diseases such as type 2 diabetes, regardless of weight status[21]. Future interventions may consider tailoring efforts to younger, male audiences and developing store-based strategies to address sugary drink consumption. Finally, our finding that nutrition education, improved access to quality produce, and more affordable healthy foods were the top strategies identified by respondents to improve the diet of neighborhood residents points to the opportunity to develop initiatives which simultaneously address multiple barriers to eating healthy including knowledge, access, and price.

A strength of this study is that our data were obtained through surveys with neighborhood residents, and therefore may offer a more general indication of shopping behaviors than surveys conducted with shoppers at specific store locations. An important limitation is the lack of generalizability of our findings. NYC is a unique environment, with high population density, limited reliance on cars, and a unique food environment featuring a high density of food retail outlets[22],[23]. Our data only reflect two Bronx neighborhoods, which may feature distinct food environment characteristics and related behaviors in comparison to other NYC neighborhoods. Additional research is needed to compare shopping behaviors across NYC neighborhoods and between NYC and other cities. Our sample had a somewhat lower proportion of Hispanic/Latino respondents and a higher proportion of African American respondents than the population estimates reported by the U.S. Census Bureau for these neighborhoods. Additionally, we are unable to draw conclusions about how shopping behaviors among our study sample relate to the specific characteristics of their neighborhood food environment in comparison with other neighborhoods.

The findings from our study have important implications for public health practitioners seeking to work with local food retailers to create a more health-promoting food environment. Our results indicate that residents of the study neighborhoods have high exposure to local food stores and support the need to work with bodegas and supermarkets to encourage healthier behaviors.

**Table 4.** Respondent Shopping Behaviors, Shop Healthy NYC Street Intercept Survey (N = 505), Bronx, NY, 2012.

	Overall	Gender		p-value	Age Group				p-value	Race/Ethnicity				p-value	Education*			p-value
		Male	Female		18–24	25–44	45–64	65+		Hispanic	Black	White	Other		Less than HS	HS Grad/GED	Any College	
	N %	N %	N %		N %	N %	N %	N %		N %	N %	N %	N %		N %	N %	N %	
Supermarket Shopping																		
Ever shops at neighborhood supermarkets	485	225	256	0.811	50	186	219	30	0.190	286	162	15	16	< 0.01	150	126	156	0.783
	96.8	97.0	96.6		92.6	98.4	96.5	96.8		97.6	96.4	83.3	100		98	96.9	96.9	
Shops at supermarkets at least once a week	301	133	165	0.471	25	114	139	23	0.099	177	98	10	11	0.057	82	81	111	0.049
	60.1	57.3	62.3		46.3	60.3	61.2	74.2		60.4	58.3	55.6	68.8		53.6	62.3	68.9	
Usual supermarket is inside respondents' neighborhood	413	190	220	0.849	43	150	190	30	0.070	245	134	14	15	0.531	132	108	128	0.285
	83.8	83.3	84		86	79.4	85.2	96.8		84.8	81.2	82.4	93.8		87.4	82.4	81	
Bodega Shopping																		
Ever shops at neighborhood bodegas	477	226	247	0.066	55	182	213	27	< 0.01	277	160	18	14	0.765	144	125	149	0.849
	94.6	96.6	92.9		100	96.3	93.4	84.4		94.2	94.7	100	93.3		94.1	94.7	93.1	
Shops at bodegas at least once a day	30	169	158	< 0.01	46	123	144	17	0.013	188	117	10	9	0.507	98	91	92	0.126
	65.5	72.2	59.4		83.6	65.1	63.2	53.1		64	69.2	55.6	60		64.1	68.9	57.5	
Most Common Fruit & Vegetable Source																		
Supermarket	376	178.0	195	0.402	43	141	168	24	0.819	219	126	13	13	0.760	116	98	117	0.741
	76	77.4	74.1		81.1	75.4	75	75		75	75.9	72.2	86.7		77.3	74.8	73.6	
Any other location	120	52.0	68	0.402	10	46	56	8	0.819	73	40	5	2	0.760	34	33	42	0.741
	24	23	25.9		18.9	24.6	25	25		25	24.1	27.8	13.3		22.7	25.2	26.4	

*Education is presented among those aged 25 and up
